# Prenatal HIV Test Uptake and Its Associated Factors for Prevention of Mother to Child Transmission of HIV in East Africa

**DOI:** 10.3390/ijerph18105289

**Published:** 2021-05-16

**Authors:** Feleke Hailemichael Astawesegn, Virginia Stulz, Kingsley E. Agho, Haider Mannan, Elizabeth Conroy, Felix Akpojene Ogbo

**Affiliations:** 1 Translational Health Research Institute (THRI), Campbelltown Campus, Western Sydney University, Penrith, NSW 2751, Australia; h.mannan@westernsydney.edu.au (H.M.); e.conroy@westernsydney.edu.au (E.C.); felgbo@yahoo.co.uk (F.A.O.); 2 School of Public Health, College of Medicine and Health Sciences, Hawassa University, Hawassa P.O. Box 1560, Ethiopia; 3 School of Nursing and Midwifery Centre for Nursing and Midwifery Research, Western Sydney University, Kingswood, NSW 2340, Australia; v.stulz@westernsydney.edu.au; 4 School of Health Sciences, Western Sydney University, Penrith, NSW 2751, Australia; K.agho@westernsydney.edu.au; 5 African Vision Research Institute, University of KwaZulu-Natal, Westville Campus, Durban 3629, South Africa; 6 General Practice Unit, Prescot Specialist Medical Centre, Welfare Quarters, Makurdi 972261, Nigeria

**Keywords:** prenatal HIV testing, prevention of mother to child HIV transmission, HIV/AIDS, East Africa

## Abstract

Identifying the socioeconomic and structural issues that act as enablers and/or barriers to HIV testing services is critical in combatting HIV/AIDS amongst mothers and children in Africa. In this study, we used a weighted sample of 46,645 women aged 15–49 who gave birth in the two years preceding the survey from the recent DHS dataset of ten East African countries. Multivariable logistic regression was used to investigate the factors associated with prenatal HIV test uptake in East Africa. The overall prenatal HIV test uptake for the prevention of mother-to-child transmission (PMTCT) of HIV was 80.8% (95% CI: 74.5–78.9%) in East Africa, with highest in Rwanda (97.9%, 95% CI: 97.2–98.3%) and lowest in Comoros (17.0%, 95% CI: 13.9–20.7%). Common factors associated with prenatal HIV test service uptake were higher maternal education level (AOR = 1.29; 95% CI: 1.10–1.50 for primary education and AOR = 1.96; 95% CI: 1.53–2.51 for secondary or higher education), higher partner education level (AOR = 1.24; 95% CI: 1.06–1.45 for primary education and AOR = 1.56; 95% CI: 1.26–1.94 for secondary or higher school), women from higher household wealth index (AOR = 1.29; 95% CI: 1.11–1.50 for middle wealth index; AOR = 1.57; 95% CL: 1.17–2.11 for rich wealth index), improved maternal exposure to the media, and increased awareness about MTCT of HIV. However, residents living in rural communities (AOR = 0.66; 95% CI: 0.51–0.85) and travelling long distances to the health facility (AOR = 0.8; 95% CI: 0.69–0.91) were associated with non-use of prenatal HIV test service in East African countries. In each East African country, factors associated with prenatal HIV test uptake for PMTCT varied. In conclusion, the pooled prenatal HIV test uptake for PMTCT of HIV was low in East Africa compared to the global target. Scaling up interventions to improve enablers whilst addressing barriers to the use of prenatal HIV test services are essential to end the HIV/AIDS epidemic in East African countries.

## 1. Introduction

Transmission of human immune virus (HIV) from an infected mother to her child during pregnancy, labor, birth, or breastfeeding is known as mother-to-child transmission (MTCT) or vertical transmission [1,2]. MTCT is the major source of HIV infection amongst children under the age of 15 and it accounts for more than 90% of children living with HIV [3]. The intervention aiming to prevent HIV transmission from mothers to their children is known as prevention of mother to child transmission of HIV (PMTCT) [4]. The preventative measures include: (i) the primary prevention of HIV infection amongst women of childbearing age; (ii) the prevention of unintended pregnancies amongst women living with HIV; (iii) the prevention of HIV transmission for women living with HIV to their infants; (iv) the provision of appropriate treatment, care, and support to mothers living with HIV, their children and families [5,6]. When PMTCT of HIV is effectively implemented, it could reduce the risk of HIV transmission to less than 1% with the use of antiretroviral treatment before, during and after pregnancy [7,8].

Substantial improvements have been implemented to reduce the burden of HIV-related deaths, morbidity, and disabilities since the onset of the global epidemic of HIV/AIDS, worldwide [9,10,11]. These improvements have been achieved through enhanced and sustained preventive strategies such as improved HIV awareness and prevention of mother-to-child transmission (PMTCT) of HIV services, increased diagnosis and testing and the use of newer antiretroviral medications, as well as targeted funding from national governments, global health organizations, and philanthropists [12,13]. The benefits of PMTCT of HIV in the community have been well documented in many low- and middle-income countries, including South Africa [14], Burkina-Faso [15] and Malawi [16].

Despite these improvements, in 2018, the United Nations Programme on HIV/AIDS (UNAIDS) estimated that approximately 1.7 million children younger than 15 years of age lived with HIV/AIDS globally [9] and the majority (90%) of those children reside in Sub-Saharan Africa (SSA) [17]. Additionally, an estimated 160,000 new HIV infections and 100,000 AIDS-related global deaths occurred amongst children under 15 years of age in 2018 [9]. Of these, 61% of new HIV infections [18] and 73% of the deaths occurred in SSA alone, reflecting the huge burden of HIV/AIDS in the African subregion [19].

In many East African countries, despite the increasing availability of free maternal and child health services (including PMTCT of HIV programs) [20,21,22], studies have shown that many countries were not achieving the universal standard of HIV testing amongst pregnant women, one of the main tenets for the PMTCT of HIV [23,24,25,26,27]. Research conducted at the national level for some East African countries has shown that a lack of achievement of universal HIV testing may be due to multiple factors. Common factors associated with the non-use of HIV testing service in Ethiopia, Kenya and Zambia included poor access to services, shortage of health professionals, poor health professionals–patient interactions, and a lack of HIV testing equipment [25,26,28]. Other factors associated with non-use of prenatal HIV testing in PMTCT of HIV included lower maternal education and young maternal age (<20 years) in Kenya and Uganda [24,26] and stigma and poor health-seeking behaviors in Zambia and Kenya [28,29].

While these country-specific studies are useful, they do not provide a comprehensive assessment of factors associated with the prenatal HIV test uptake in PMTCT of HIV in East Africa. Primarily, most of the existing studies were conducted amongst women visiting health facilities, and pregnant women who did not have access to health facilities were excluded [23,24,25,26]. Secondly, most studies are lacking a theoretical framework such as Andersen’s behavioral model for synthesizing the multitude of factors found to be associated with prenatal HIV testing for PMTCT of HIV. The lack of theoretical frameworks to guide variables classification and method of analysis to identify factors affecting prenatal HIV testing service utilization has made it difficult to collate findings across studies and to identify appropriate strategies to improve the service uptake. Thirdly, some studies were conducted more than a decade ago, and the findings may not reflect the current socioeconomic and health context of the country [26].

Therefore, the identification and implementation of relevant interventions that address socioeconomic and structural issues that deter women from accessing PMTCT services remain key strategies to improve access to HIV prevention, testing and treatment services [30]. Accordingly, this study used the most recent Demographic and Health Survey (DHS) of 10 countries to conduct a pooled analysis of prenatal HIV test uptake and associated factors in the prevention of MTCT of HIV in East Africa. We used recent data from 10 countries in WHO regions of East Africa [31,32] with completed DHS datasets between 2011 and 2017. For the remaining 11 East African countries, the DHS datasets were not complete for this period. Included in the analysis were Burundi, Comoros, Ethiopia, Kenya, Malawi, Rwanda, Mozambique, Uganda, Zambia, and Zimbabwe.

Evidence from this study will be useful for in-country stakeholders to guide PMTCT efforts to increase access to HIV testing, treatment, care and support services amongst women in the Eastern Africa region. The information will also be helpful as each country in the region makes appropriate efforts towards achieving the UNAIDS 95-95-95 goals [33].

## 2. Methods and Materials

### 2.1. Study Design and Data Sources

This analytical cross-sectional study used data from the DHS program [34]. The DHS are nationally representative household surveys that provide data for a wide range of monitoring and impact evaluation indicators in the areas of population, health and nutrition [35]. The information on the HIV testing during antenatal care and birth in the two years preceding the survey for each woman in the sample was found in the women’s individual records of DHS.

The most recent data from 10 countries in WHO regions of East Africa [31,32] with completed DHS datasets between 2011 and 2017 were included in this study. This period covered the transition between the completion of the Millennium Development Goals (MDG’s) and the commencement of the Sustainable Development Goals (SDGs) and this evidence will appreciate the achievement of the MDGs and will be useful as a baseline for the SDGs. Included countries in the analysis were Burundi (DHS, 2016–2017), Comoros (DHS, 2012), Ethiopia (DHS, 2016), Kenya (DHS, 2014), Malawi (DHS, 2015–2016), Mozambique (DHS, 2011), Rwanda (DHS, 2014–2015), Uganda (DHS, 2016), Zambia (DHS, 2013–2014), and Zimbabwe (DHS, 2015). Pooled DHS datasets from 10 East African countries were done by creating a country-specific cluster and country-specific strata. Accordingly, in the present study, we used a total weighted sample of 46,645 women age 15–49 years who gave birth in the two years preceding the survey to determine the pooled magnitude and determinants of prenatal HIV test uptake across East Africa countries.

The DHS employed a cross-sectional study design with a stratified two-stage sampling strategy, where country was divided into enumeration areas (clusters) based on the census frames in the country, and then, households were randomly selected within each cluster. Furthermore, since the DHS surveys were intended to address household-based health issues, strata for urban and rural households were used for the selection of respondents. The DHS follows a standard procedure of data collection and presentation (similar questionnaires) and uses the same definition of terms. The DHS data were collected by the country-specific department of health and population, in collaboration with Inner City Fund (ICF) International using standardized household questionnaires. The detailed methodology of the survey design, sample selection, survey tools and data collection are described elsewhere [36,37].

### 2.2. Outcome Variables

In this study, prenatal HIV test uptake was measured as the proportion of women who tested for HIV and received their HIV test result during pregnancy, consistent with the PMTCT strategy [38,39]. Therefore, for this study prenatal HIV test uptake was coded as “1” if a woman was tested for HIV and received the HIV test result during antenatal care or before birth, otherwise coded as “0” if the woman did not test for HIV or tested but did not receive the test result during antenatal care or before birth.

### 2.3. Explanatory Variables

We adapted the most recent/the fourth phase/behavioral model of health service utilization by Ronald M. Andersen for this study [40]. It is a well-validated and most widely adopted theoretical framework that permits systematic identification of factors that influence individual decisions to use or not to use available health care services [40]. Several studies have used this conceptual model to study health care utilization [41,42,43,44]. The selection of study variables to be included in this study was done based on the purpose of this research, previously published literature from low- and middle-income countries [45,46], and the availability of information regarding the relevant variables. Study variables were categorized into community levels, predisposing, enabling and need factors based on the modified Andersen model [40]. Accordingly, the following were variables extracted from the DHS data and their classification for this study.

Community level factors reflect the contextual or environmental characteristics affecting the use of health services. Included are place of residence (categorized as rural or urban) and country of residence (Burundi, Comoros, Ethiopia, Kenya, Malawi, Rwanda, Tanzania, Mozambique, Uganda, Zambia). Burundi was selected as the reference country as it is the first country on the list of East African countries.

Predisposing factors reflect the individuals’ characteristics that influence the propensity to use health services before illness onset. It consists of maternal age (classified as 15–24, 25–34, and 35–49 years), maternal and partner educational level (categorized as no education, primary or secondary and above education) and employment status (categorized as not working, formal employment and non-formal employment). Women’s history of any sexual violence by her husband/partner (categorized as yes or no), women listening to the radio (categorized as yes or no), watching television (categorized as yes or no) and reading magazines or newspapers (categorized as yes or no) were the other predisposing factors.

Enabling factors encompass personal or community resources that can promote or inhibit access to health services. These included wealth index, the household wealth index for the pooled dataset, which was constructed using the ‘hv271′ variable. The ‘hv271′ is a household’s wealth index value generated by the product of standardized scores (z-scores) and factor coefficient scores (factor loadings) of wealth indicators [47]. Within the household wealth index categories, the bottom 20% of households were arbitrarily referred to as the poorest households, and the top 20% as the wealthiest households and they were grouped into poor, middle and rich based on previously published studies [48]. Women’s involvement in household decisions is derived from four different household decisions including decisions to seek health care, decisions on large household purchases, decisions on what to do with the money the husband earns and decisions to visit family/relatives. It is categorized as involved in the household decision if a woman decides on one or more household decisions, otherwise not involved. Perceived distance to health facilities was dichotomously categorized as challenging or not. Women’s awareness about MTCT of HIV during pregnancy, awareness of MTCT during birth and awareness of MTCT during breastfeeding were all classified as yes or no.

Need factors represent the potential needs of health service use according to the women’s perceived or evaluated health status which includes women’s intention for the pregnancy (categorized as desired pregnancy if the pregnancy is wanted, otherwise unwanted pregnancy).

### 2.4. Statistical Analyses

The analysis is based on pooled DHS datasets from 10 East African countries by creating country-specific clustering and country-specific strata using similar methods employed by Agho et al. [49]. Throughout the analysis population-level weight was used to adjust for the imbalance of country-specific populations across East Africa countries. Descriptive statistics such as percentage, frequency counts, the prevalence of prenatal HIV test uptake and its 95% confidence intervals were conducted for all East African countries and each country.

Logistic regression models were used to investigate the influence of the study factors on prenatal HIV test uptake in PMTCT of HIV services after adjusting for country-specific cluster and population level weights using the “svy: logistic” command. Four-stage modeling using the adapted Andersen’s behavioral model of health service utilization was executed to determine the adjusted odds ratios and compare the relative influence of the four kinds of factors on prenatal HIV test uptake for PMTCT of HIV services [40]. Community level factors (place of residence and country of residence) were entered in the first stage model. In the second stage model, community level factors and predisposing factors (maternal age, maternal education, partner education, history of sexual violence, maternal employment, women listen to the radio, watch television, and read newspapers) were included. In the third stage, the second stage model was added to enabling factors (household wealth index, women’s involvement in household decisions, health facility distance, aware MTCT during pregnancy, aware MTCT during birth and aware MTCT during breastfeeding) followed by the fourth or final stages, in which the third stage model was then added to the need factors (desire for the pregnancy). 

Adjusted odds ratios (AORs) with their 95% confidence intervals (CIs) and *p*-value < 0.05 were estimated to determine the presence of association between study factors and prenatal HIV test uptake. All statistical analyses were conducted using STATA version 14.2 (Stata Corp, College Station, TX, USA).

## 3. Results

### 3.1. Characteristics of the Study Participants in East Africa

A weighted total sample of 46,645 eligible women aged 15–49 years who had birthed in the previous two years before the surveys were used in the study. Almost half (45%) of the women were aged 25–34 years, while almost two-fifths (37%) were in the age range of 15–24 years (Supplementary Table S1). Almost three-quarters (77%) of women resided in rural areas, and half of the women in the study had attained a primary level of education. More than half (56%) of the women were not involved in household decision-making processes and did not perceive distance to the health facility as challenging (55%).

### 3.2. The Proportion of Women Who Used HIV Test Services for PMTCT of HIV in East African Countries by Each Study Factor

The analysis showed that more than three-quarters (80.8%, 95% CI: 79.8–81.8%) of women in East African countries used prenatal HIV test services for PMTCT of HIV between 2011 and 2017.

**Figure 1:**
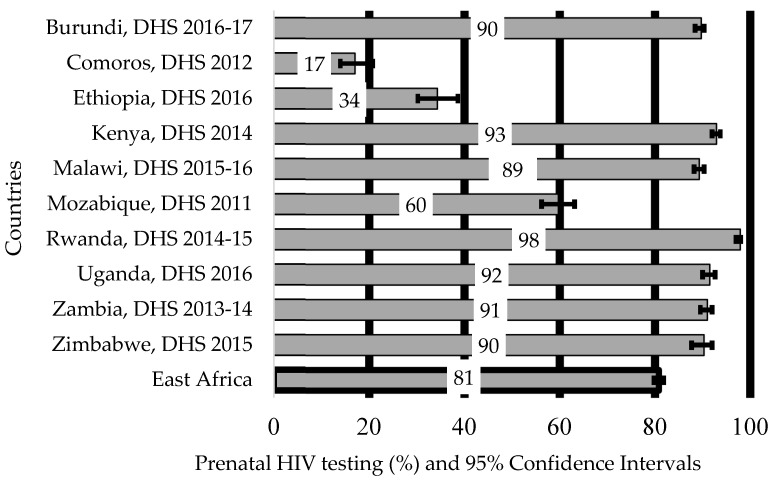
Women who used prenatal HIV testing services for prevention of mother to child transmission of HIV in East African countries (2011–2017).

The highest proportion of women who used prenatal HIV test services for PMTCT of HIV was recorded in Rwanda (97.9%, 95% CI: 97.2–98.3%), followed by Kenya (92.9%, 95% CI: 92.1–93.7%) and Uganda (91.5%, 95% CI: 90.2–92.6%). In contrast, countries with the lowest proportion of HIV test services were Comoros (17.0%, 95% CI: 13.9–20.7%), Ethiopia (34.3%, 95% CI: 30.3–38.6%) and Mozambique (59.7%, 95% CI: 56.2–63.1%) (Figure 1).

**Figure 2:**
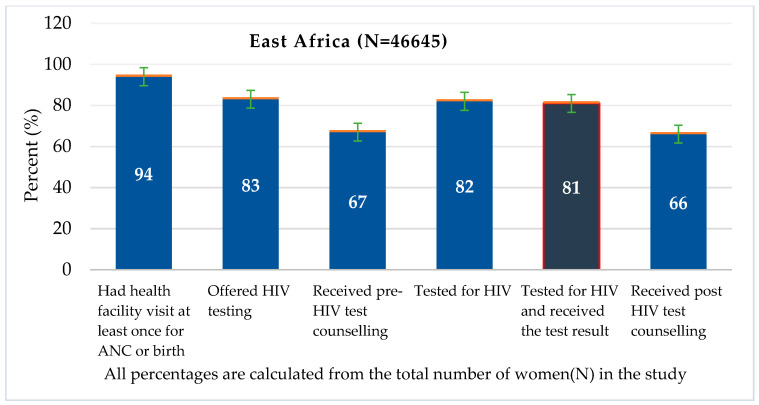
Health facility visits during pregnancy and HIV test services uptake in East Africa.

This study also depicted the percentages of those women who attended facility visits during pregnancy and received HIV testing in East Africa to identify missed opportunities. Of all pregnant women who participated in the survey, 94.4% (95% CI: 93.7–95%) had visited health facilities at least once for antenatal clinic or birth services, 82.5% (95% CI: 81.5–83.5%) had been offered HIV testing, 66.8% (95% CI: 65.8–67.8%) received pre-HIV test counselling and 66.3% (95% CI: 65.2–67.4%) received post HIV test counselling (Figure 2). The proportion of women who visited a health facility and who received HIV testing and counselling services varies across countries. The highest missed opportunities for prenatal HIV testing were observed in Comoros (74%, 95% CI: 72–75%) followed by Ethiopia (34%, 95% CI: 33.8–34.5%), (Supplementary Figure S1).

Table 1 shows prenatal HIV test uptake for PMTCT of HIV by study factors in each and all East African countries. Across East African countries, women who resided in rural areas of Burundi (81.0%), Malawi (76.7%), Rwanda (80.7%) and Uganda (70.9%) had the highest proportion of services use. Amongst women who attained secondary and higher education, those who resided in Zambia (34.0%) and Zimbabwe (62.5%) reported the highest level of usage of HIV test services during pregnancy, whilst the lowest was reported in Burundi (11.2%), Comoros (8.6%) and Ethiopia (6.8%) (Table 1).

### 3.3. Factors Associated with Prenatal HIV Testing for PMTCT of HIV in East African Countries

The present study indicated that women who resided in rural communities were less likely to utilize HIV test services for PMTCT of HIV compared to their urban counterparts (AOR = 0.66; 95% CI: 0.51–0.85). Compared to Burundi, the odds for using prenatal HIV test services were lower in Comoros, Ethiopia, Mozambique, Uganda, Zambia and Zimbabwe (AOR = 0.007; 95% CI: 0.005–0.01 for Comoros; AOR = 0.04; 95% CI: 0.03–0.05 for Ethiopia; AOR = 0.15; 95% CI: 0.11–0.20 for Mozambique; AOR = 0.61; 95% CI: 0.46–0.82 for Uganda; AOR = 0.54; 95% CI: 0.39–0.73 for Zambia and AOR = 0.42; 95% CI: 0.28–0.64 for Zimbabwe). Rwandan women were two times more likely to use HIV testing services during pregnancy compared to those who resided in Burundi (AOR = 2.30; 95% CI: 1.29–4.08).

Our study showed that women who attained at least a primary level of education were more likely to use HIV testing services for PMTCT of HIV compared to those with no formal schooling (AOR = 1.29; 95% CI: 1.10–1.50 for primary education and AOR = 1.96; 95% CI: 1.53–2.51 for secondary or higher education). Women whose partners had at least primary education were more likely to use HIV testing services for PMTCT of HIV compared to their counterparts (AOR = 1.24; 95% CI: 1.06–1.45 for primary education and AOR = 1.56; 95% CI: 1.26–1.94 for secondary or higher school). Women who read magazines, watched television and listened to the radio were more likely to use HIV testing services compared to their counterparts (AOR = 1.31, 95% CI: 1.04, 1.65 for magazine; AOR = 1.46, 95% CI: 1.20, 1.79 for television; AOR = 1.13,95% CI: 1.01–1.29 for radio).

Women who lived in wealthier households had a greater chance of using HIV testing services during pregnancy compared to those who lived in poorer households (AOR = 1.29; 95% CI: 1.11–1.50 for middle wealth index; AOR = 1.57; 95% CL: 1.17–2.11 for rich wealth index). Women who perceived the distance to health facilities as challenging had a lower chance of using HIV testing services compared to their counterparts (AOR = 0.8; 95% CI: 0.69–0.91). Additionally, women who had an awareness about perinatal MTCT of HIV were more likely to be tested for HIV (AOR = 1.73; 95% CI: 1.42–2.10 during birth; AOR = 1.41; 95% CI: 1.16–1.71 during breastfeeding period) (Table 2).

The study showed various associations between the study factors and prenatal HIV test uptake for PMTCT of HIV in each East African country (Supplementary Table S2). For example, history of sexual violence, older maternal age, and unwanted pregnancy were negatively associated with using HIV testing services for PMTCT of HIV, whereas women who were involved in household decision making were positively associated with using HIV testing services for PMTCT of HIV.

## 4. Discussion

Universal HIV testing and counselling is the first step and the entry point for pregnant women to receive other PMTCT services. WHO recommended that countries implement an opt-out strategy or provider-initiated HIV testing during antenatal clinic visits to increase the likelihood of HIV testing [50]. Therefore, in this article, we assessed the proportion of pregnant women who had been tested for HIV and received the test result as well as factors associated with HIV testing as part of PMTCT services in East Africa. This study showed that the overall prenatal HIV testing for PMTCT of HIV in East African countries was 80.8%; highest in Rwanda (97.9%) and lowest in Comoros (17.0%). Across East African countries, common factors associated with the use of prenatal HIV testing for PMTCT of HIV were higher parental education, higher household wealth index, improved maternal exposure to the media and increased awareness about MTCT. However, residents in rural communities and those having long distances to travel to the health facility were associated with non-use of the services in East African countries. In each East African country factors associated with the utilization of prenatal HIV testing for PMTCT of HIV varied in the analyses.

This study showed that differences in the proportion of HIV testing uptake in East African countries exist, ranging from 17.0% in Comoros to 97.9% in Rwanda. Additionally, in comparison to Burundian women, those who reside in Comoros, Ethiopia, Mozambique, Uganda, Zambia and Zimbabwe were less likely to use HIV test services during pregnancy. While, Rwandan women were more likely to do so. These variations across countries may be explained by differences in sociodemographic characteristics, health systems infrastructure and timely adoption and implementation of the WHO PMTCT guidelines (an “opt-out” strategy) [51,52], as well as the progressive laws and policy frameworks that may have facilitated the prenatal HIV testing [53,54,55]. In countries with lower HIV testing uptake, the following reasons may have played a role; poor quality of PMTCT services [28], lack of resources such as HIV test kit stock-outs [56,57], low awareness of MTCT and stigma towards HIV positive women [27,29]. Prominently, increased missed opportunity in Comoros (74%) and Ethiopia (35%) evidenced by this study due to weak integration of HIV testing with maternal services [4,58] could also be responsible for lower HIV testing uptake.

This study has identified that rural residents were less likely to utilize HIV testing services, which corroborates with previous studies [59,60]. It could be argued that lower HIV testing service use amongst women living in rural areas, could be related to unequal access to society’s resources that include education, employment and health care services [60,61,62,63,64] and existing systematic socioeconomic disparities between urban–rural dwellers. Additionally, increased stigma and discrimination in rural communities [65] and shortages of qualified health professionals and supplies in the rural health facilities [60,66] may lead to lower utilization of HIV testing services amongst pregnant women from rural areas. This suggests that strategies targeting rural areas must be strengthened to reduce barriers to prenatal HIV testing for PMTCT of HIV.

It has been identified that higher maternal and partner educational status were significantly associated with the likelihood of HIV testing services use during pregnancy. This finding aligns with studies in Zambia [28], Uganda [46], Kenya [26] and Ethiopia [65]. This is explained by educated partners having better access to health information and that they are more likely to realize the benefits of HIV testing during pregnancy to prevent HIV transmission and therefore more likely to seek, accept and test for HIV. Educated partners are more likely to have a planned pregnancy [67,68] and this may enable them to think carefully about what the pregnancy requires so that they can mobilize the resources that are needed for maternal services, including PMTCT services. Meaningfully, more than 90% of women in this study reported that their pregnancy was planned and of those, approximately 75% had been tested for HIV. Furthermore, educated women are more likely to make decisions to seek health care [69] and reside in urban areas where there is easier access to health facilities that provide quality PMTCT of HIV services [63,66].

Maternal awareness regarding mother to child HIV transmission during birth and breastfeeding was found to be an independent predictor of prenatal HIV testing. This is consistent with previous findings from Ethiopia, South Africa, Tanzania and Botswana [27,59,70,71] which identified recognizing the risk of HIV transmission from mother to infant at the time of pregnancy, birth or breastfeeding being the reason for having been tested for HIV. Similarly, exposure to the media (radio/TV/magazine) was also associated with prenatal HIV testing for PMTCT of HIV due to women being exposed to different electronic and print media that increases awareness about the benefits of PMTCT services.

Our study also demonstrates that women from higher wealth index households were more likely to have been tested for HIV during pregnancy, which is consistent with findings from studies in Kenya, Ethiopia, South Africa and Uganda [26,52,65,72]. Despite maternal services being free of charge in most East African countries, distances to health care facilities and the transportation costs to access health services could be challenges for poorer citizens. Furthermore, this study found that women who perceived the long distances to health facilities as challenging were less likely to use services, which is consistent with previous research in Uganda, Ethiopia, and South Africa [23,24,72,73]. This urges all responsible governmental and non-governmental organizations to make the services more accessible to pregnant women. Improving referral systems for HIV testing and scaling up community-based/home-based HIV counselling and testing could be a better alternative to reach pregnant women who cannot access health facility-based counselling and testing. Home-based HIV testing and counselling approaches are recommended in developing countries with lower antenatal clinic services [45], which could increase the uptake of these services amongst pregnant women [74,75].

Findings in this study should be interpreted considering the inherent limitations of this study. In the first instance, since DHS uses a cross-sectional study design, a clear temporal association cannot be established between the study factors and prenatal HIV test uptake in PMTCT of HIV. Secondly, there is a possibility of recall bias as there is a gap between the time of services use and the timing of data collection. Moreover, the information on the study factors and outcome variable was based on self-reporting. The strengths included the analyses being based on a national level community-based survey and this provides representative samples (to avoid selection bias); therefore, findings can be generalized for the regions. The results are comparable across the East African countries as all the variables used were similarly described across countries. This study provides relevant contextual evidence for different stakeholders since we used a large sample size that represents East African countries.

## 5. Conclusions

Our study showed that the overall prenatal HIV testing uptake for PMTCT of HIV was low (80.8%) in 10 selected East African countries compared to the global target of 95% by the year 2030, respectively. Across these East African countries, the common factors associated with the utilization of prenatal HIV testing services were higher parental education, higher household wealth index, improved maternal exposure to the media and increased awareness about MTCT. However, residents in rural communities and those who perceive long distances to the health facilities as challenging were associated with the non-use of HIV testing services for PMTCT in these East African countries. These findings indicate the need to improve the implementation of PMTCT of HIV services in such a way that solutions should address the disadvantaged subpopulation group; such as designing outreach HIV testing services to reach rural residents and the poor who are unable to visit health facilities. Furthermore, countries should work more to enhance universal primary education for all and create awareness on the prevention of mother-to-child transmission of HIV services through various outlets of media.

## Figures and Tables

**Table 1:** Percentage of prenatal HIV test service user for PMTCT of HIV by study factors in East African countries (N = 46,645; Demographic and Health Survey, 2011–2017).

	Burundi	Comoros	Ethiopia	Kenya	Malawi	Mozambique	Rwanda	Uganda	Zambia	Zimbabwe	Total
	Yes ^#^ n (%)	Yes ^#^ n (%)	Yes ^#^ n (%)	Yes ^#^ n (%)	Yes ^#^ n (%)	Yes ^#^ n (%)	Yes ^#^ n (%)	Yes ^#^ n (%)	Yes ^#^ n (%)	Yes ^#^ n (%)	Yes ^#^ n (%)
**Community level factors**											
Residency											
Urban	471 (8.7)	96 (7.4)	410 (9.5)	2530 (34.4)	848 (12.6)	1050 (21.4)	554 (17.1)	1214 (20.6)	1656 (32.6)	653 (26.6)	16,238 (20.3)
Rural	4384 (81.0)	125 (9.6)	1069 (24.8)	4306 (58.5)	5131 (76.7)	1884 (38.3)	2612 (80.7)	4185 (70.9)	2962 (58.4)	1563 (63.7)	38,289 (60.5)
**Predisposing factors**											
Maternal age											
15–24	1255 (23.2)	68 (5.3)	453 (10.5)	2688 (36.5)	2717 (40.6)	1223 (24.9)	852 (26.3)	2290 (38.8)	1816 (35.8)	882 (36.0)	14,244 (30.6)
25–34	2481 (45.9)	111 (8.5)	766 (17.8)	3186 (43.3)	2361 (35.3)	1250 (25.5)	1634 (50.5)	2306 (39.1)	1998 (39.4)	1029 (41.9)	17,120 (36.7)
35–49	1117 (20.6)	42 (3.2)	260 (6.0)	963 (13.1)	897 (13.4)	455 (9.3)	680 (21.0)	799 (13.6)	804 (15.8)	305 (12.4)	6321 (13.5)
Maternal education											
No education	2096 (38.7)	64 (4.9)	612 (14.2)	615 (8.4)	673 (10.0)	817 (16.6)	417 (12.9)	499 (8.5)	424 (8.4)	28 (1.1)	6244 (13.4)
Primary	2155 (39.8)	46 (3.5)	571 (13.3)	3775 (51.3)	3983 (59.5)	1552 (31.6)	2276 (70.3)	3200 (54.2)	2464 (48.6)	654 (26.7)	20,676 (44.3)
Secondary and higher	603 (11.2)	111 (8.6)	296 (6.8)	2445(33.2)	1323 (19.8)	565 (11.5)	474 (14.6)	1699 (28.8)	1725 (34.0)	1534 (62.5)	10,777 (23.1)
Maternal occupation											
Not working	386 (7.1)	123 (9.8)	776 (18.0)	1146 (32.4)	1846 (27.6)	1728 (53.0)	254 (7.8)	1001 (17.0)	2084 (41.6)	1216 (49.7)	10,560 (25.7)
Professional work	457 (8.5)	40 (3.2)	321 (7.4)	547 (15.5)	515 (7.7)	413 (12.6)	417 (12.9)	1161 (19.7)	971 (19.4)	687 (28.1)	5528 (13.5)
Nonprofessional work	4012 (74.1)	40 (3.2)	382 (8.9)	1584 (44.9)	3619 (54.0)	76 (2.3)	2494 (77.1)	3234 (54.8)	1503 (30.0)	306 (12.5)	17,249 (42.0)
Partner education											
No education	1589 (32.3)	39 (3.1)	461 (11.3)	220 (6.3)	522 (9.3)	662 (14.3)	449 (15.5)	399 (8.0)	360 (8.0)	20 (1.0)	4723 (12.4)
Primary	2270 (46.2)	43 (3.5)	545 (13.4)	1500 (47.1)	2735 (48.9)	1359 (29.2)	2007 (69.5)	2342 (47.1)	1605 (35.5)	418 (20.0)	14,825 (38.9)
Secondary and higher	559 (11.4)	117 (9.4)	393 (9.6)	1237 (38.9)	1803 (32.2)	697 (15.0)	372 (12.9)	1804 (36.3)	2141 (47.3)	1449 (69.3)	10,574 (27.7)
History of Sexual violence											
No	2513 (68.0)	168 (16.3)	549 (29.4)	1151 (83.2)	1372 (73.5)	1437 (53.2)	654 (89.8)	2267 (72.0)	2927 (76.4)	1522 (80.6)	14,560 (65.7)
Yes	799 (21.6)	1 (0.1)	62 (3.3)	129 (9.3)	298 (16.0)	119 (4.4)	58 (7.9)	629 (19.9)	538 (14.1)	175 (9.3)	2808 (12.7)
Read newspapers or magazines											
No	4643 (85.8)	154 (11.9)	1266 (29.4)	4753 (64.6)	4970 (74.2)	2503 (50.9)	2479 (76.7)	4321 (73.2)	3333 (65.7)	1413 (57.6)	29,834 (63.9)
Yes	212 (3.9)	67 (5.2)	213 (4.9)	2081 (28.3)	1010 (15.1)	431 (8.8)	682 (21.1)	1079 (18.3)	1285 (25.3)	803 (32.7)	7862 (16.9)
Listened to the radio											
No	2664 (49.2)	92 (7.1)	888 (20.6)	1317 (17.9)	3127 (46.7)	977 (19.9)	576 (17.8)	1429 (24.2)	1932 (38.1)	980 (39.9)	13,982 (29.9)
Yes	2191 (40.5)	129 (9.9)	591 (13.7)	5516 (75.0)	2852 (42.6)	1957 (39.8)	2590 (80.0)	3971 (67.3)	2686 (52.9)	1235 (50.4)	23,719 (50.9)
Watched television											
No	4488 (82.3)	51 (3.9)	986 (22.9)	3813 (51.9)	5028 (75.1)	1883 (38.3)	1977 (61.2)	3866 (65.5)	2973 (58.6)	1352 (55.1)	26,417 (56.6)
Yes	366 (6.8)	170 (13.1)	493 (11.4)	3018 (41.1)	951 (14.2)	1051 (21.4)	1184 (36.7)	1533 (26.0)	1645 (32.4)	864 (35.2)	11,276 (24.2)
**Enabling factors**											
Household wealth index											
Poor	2090 (38.6)	53 (4.1)	388 (9.0)	2888 (29.3)	2826 (42.2)	961 (19.6)	1421 (43.9)	2278 (38.6)	2095 (41.3)	964 (39.3)	15,965 (34.2)
Middle	1938 (35.8)	111 (8.6)	612 (14.2)	2507 (34.1)	2176 (32.5)	1314 (26.7)	1176 (36.3)	1989 (33.7)	1805 (35.6)	912 (37.2)	14540 (31.2)
Rich	826 (15.3)	57 (4.3)	479 (11.1)	1441 (19.6)	977 (14.6)	660 (13.4)	569 (17.6)	1132 (19.2)	717 (14.1)	340 (13.8)	7198 (15.4)
Household decision making											
Not Involved	2155 (44.0)	143 (12.3)	412 (10.1)	1744 (59.8)	3156 (57.6)	1390 (33.7)	1110 (42.5)	3055 (62.2)	2054 (50.4)	678 (32.4)	15,899 (43.7)
Involved	2250 (46.0)	54 (4.6)	986 (24.2)	970 (33.2)	1810 (33.0)	1002 (24.3)	1453 (55.6)	1437 (29.2)	1660 (40.8)	1205 (57.6)	12,827 (35.3)
Health facility distance											
Challenging	1590 (29.4)	98 (7.5)	635 (14.7)	841 (23.8)	3379 (50.5)	1499 (30.5)	722 (22.3)	2188 (37.1)	1983 (39.1)	847 (34.5)	13,781 (32.2)
Not challenging	3265 (60.3)	123 (9.5)	844 (16.6)	2448 (69.1)	2601 (38.9)	1435 (29.2)	2444 (75.5)	3212 (54.4)	2634 (51.9)	1368 (55.8)	20,375 (47.6)
Aware MTCT during pregnancy											
No	657 (12.5)	78 (6.3)	343 (8.8)	3173 (22.5)	1153 (17.6)	479 (10.0)	992 (30.7)	1558 (26.5)	1666 (32.9)	174 (7.2)	10,273 (22.5)
Yes	4198 (79.9)	143 (11.5)	1136 (29.2)	3660 (60.1)	4826 (73.8)	2455 (50.0)	2172 (67.2)	3841 (65.2)	2950 (58.4)	2042 (84.1)	27,423 (60.1)
Aware MTCT during birth											
No	246 (4.7)	100 (8.1)	286 (7.4)	1428 (19.5)	898 (13.7)	463 (9.6)	100 (3.1)	340 (5.8)	456 (9.0)	208 (8.6)	4524 (9.9)
Yes	4609 (87.7)	121 (9.7)	1193 (30.6)	5408 (73.8)	5081 (77.7)	2471 (51.5)	3066 (94.8)	5060 (85.9)	4161 (82.3)	2008 (82.7)	33,177 (72.7)
Aware MTCT during breastfeeding											
No	436 (8.3)	90 (7.3)	226 (5.8)	691 (9.4)	536 (8.2)	335 (7.0)	162 (5.0)	513 (8.7)	343 (6.8)	306 (12.6)	3639 (8.0)
Yes	4419 (84.1)	131 (10.5)	1253 (32.2)	6145 (83.8)	5444 (83.3)	2599 (54.0)	3004 (92.9)	4886 (82.9)	4273 (84.6)	1909 (78.7)	34,062 (74.6)
**Need Factors**											
A desire for the pregnancy											
Wanted pregnancy	4387 (81.1)	197 (15.2)	1407 (32.6)	2946 (83.2)	5326 (79.6)	2828 (57.6)	2752 (85.1)	4916 (83.3)	4332 (85.4)	2041 (83.2)	31,132 (72.7)
Unwanted pregnancy	468 (8.6)	24 (1.9)	71 (1.7)	345 (9.7)	654 (9.8)	106 (2.2)	413 (12.8)	484 (8.2)	283 (5.6)	175 (7.1)	3022 (7.1)

Yes ^#^ n Weighted number of women who tested for HIV during pregnancy; % = weighted proportions of women in each East African country who were tested for HIV; N = total weighted number of eligible women by study factors in East African countries.

**Table 2:** Factors associated with prenatal HIV testing for PMTCT of HIV in East African countries (DHS, 2011–2017).

Characteristic	Model 1				Model 2				Model 3				Model 4			
	AOR	95%CI		*p* Value	AOR	95%CI		*p* Value	AOR	95%CI		*p* Value	AOR	95%CI		*p* Value
** Community level factors**																
**Countries**																
Burundi	1.00				1.00				1.00				1.00			
Comoros	0.01	0.01	0.02	<0.001	0.007	0.005	0.01	<0.001	0.007	0.005	0.01	<0.001	0.007	0.005	0.01	<0.001
Ethiopia	0.05	0.04	0.06	<0.001	0.05	0.03	0.06	<0.001	0.04	0.03	0.05	<0.001	0.04	0.03	0.05	<0.001
Kenya	1.19	0.99	1.42	0.058	0.75	0.55	1.01	<0.061	0.71	0.50	1.00	0.055	0.73	0.51	1.03	0.078
Malawi	0.92	0.78	1.09	0.382	0.71	0.54	0.93	0.014	0.72	0.53	0.99	0.043	0.73	0.53	1.03	0.06
Mozambique	0.13	0.11	0.16	<0.001	0.17	0.13	0.23	<0.001	0.15	0.11	0.20	<0.001	0.15	0.11	0.20	<0.001
Rwanda	4.91	3.62	6.66	<0.001	3.18	1.79	5.64	<0.001	2.28	1.28	4.05	0.005	2.30	1.29	4.08	0.004
Uganda	1.11	0.90	1.36	0.296	0.74	0.57	0.96	0.023	0.61	0.45	0.81	0.001	0.61	0.46	0.82	0.001
Zambia	0.93	0.75	1.15	0.514	0.59	0.45	0.77	<0.001	0.54	0.40	0.73	<0.001	0.54	0.39	0.73	0.001
Zimbabwe	0.90	0.67	1.21	0.505	0.39	0.26	0.57	<0.001	0.42	0.28	0.64	<0.001	0.42	0.28	0.64	0.001
**Residence**																
Urban	1.00				1.00				1.00				1.00			
Rural	0.28	0.24	0.33	<0.001	0.53	0.42	0.66	<0.001	0.66	0.51	0.85	0.002	0.66	0.51	0.85	0.002
** Predisposing factors**																
**Maternal** **age**																
15–24					1.00				1.00				1.00			
25–34					1.02	0.90	1.15	0.752	0.92	0.80	1.05	0.245	0.93	0. 81	1.07	0.324
35–49					1.03	0.88	1.22	0.650	0.89	0.74	1.06	0.223	0.94	0.78	1.13	0.523
**Maternal** **education**																
No education					1.00				1.00				1.00			
Primary					1.42	1.23	1.63	<0.001	1.29	1.10	1.50	0.001	1.29	1.10	1.50	<0.001
Secondary and higher					2.50	1.99	3.13	<0.001	1.97	1.54	2.52	<0.001	1.96	1.53	2.51	<0.001
**Maternal** **occupation**																
Not working					1.00				1.00				1.00			
Professional work					1.05	0.87	1.27	0.569	0.90	0.74	1.10	0.329	0.90	0.74	1.09	0.294
Nonprofessional work					1.08	0.91	1.27	0.347	1.02	0.85	1.22	0.762	1.02	0.85	1.22	0.785
**Partner education**																
No education					1.00				1.00				1.00			
Primary					1.29	1.12	1.49	<0.001	1.24	1.05	1.45	0.008	1.24	1.06	1.45	0.007
Secondary and higher					1.65	1.36	2.01	<0.001	1.55	1.25	1.93	<0.001	1.56	1.26	1.94	<0.001
**History of Sexual violence**																
No					1.00				1.00				1.00			
Yes					0.92	0.79	1.07	0.306	0.92	0.78	1.09	0.366	0.93	0.78	1.10	0.408
**Read newspapers or magazines**																
No					1.00				1.00				1.00			
Yes					1.29	1.05	1.60	0.014	1.31	1.04	1.65	0.020	1.31	1.04	1.65	0.021
**Listened to the radio**																
No					1.00				1.00				1.00			
Yes					1.25	1.11	1.41	<0.001	1.10	0.97	1.26	0.121	1.13	1.01	1.29	0.047
**Watched television**																
No					1.00				1.00				1.00			
Yes					1.67	1.39	2.01	<0.001	1.46	1.19	1.79	<0.001	1.46	1.20	1.79	<0.001
** Enabling factors**																
**Household wealth index**																
Poor									1.00				1.00			
Middle									1.29	1.11	1.50	0.001	1.29	1.11	1.50	0.001
Rich									1.57	1.16	2.11	0.003	1.57	1.17	2.11	0.003
**Health facility distance**																
Challenging									0.79	0.69	0.90	0.001	0.80	0.69	0.91	0.001
Not challenging									1.00				1.00			
**Household Decision making**																
Not involved									0.88	0.78	1.00	0.065	0.88	0.78	1.01	0.072
Involved									1.00				1.00			
**Aware of MTCT during pregnancy**																
No									1.00				1.00			
Yes									0.87	0.75	1.01	0.073	1.19	1.00	1.41	0.06
**Aware of MTCT during** **birth**																
No									1.00				1.00			
Yes									1.74	1.43	2.11	<0.001	1.73	1.42	2.10	<0.001
**Aware of MTCT during breastfeeding**																
No									1.00				1.00			
Yes									1.40	1.16	1.70	<0.001	1.41	1.16	1.71	<0.001
** Need factors**																
**Desire for pregnancy**																
Wanted pregnancy													1.00			
Unwanted pregnancy													0.79	0.63	1.04	0.117

Note: AOR = adjusted odds ratio; CI = confidence interval.

## Data Availability

The data used in this article are available to download for research purposes upon registration; https://www.dhsprogram.com/data/available-datasets.cfm (accessed on 29 January 2020).

## Supplementary Materials

The following are available online at https://www.mdpi.com/article/10.3390/ijerph18105289/s1, Supplementary Figure S1: Health facility visits during pregnancy and HIV testing services in East Africa countries. Supplementary Table S1: Characteristics of the study participants in each and all East African countries (DHS, 2011–2017). Supplementary Table S2: Adjusted OR (95% CI) of factors associated with prenatal HIV test uptake for PMTCT of HIV among mothers aged 15–49 years in each East African country.
