# Supplementary material for: Prenatal HIV Test Uptake and Its Associated Factors for Prevention of Mother to Child Transmission of HIV in East Africa
Source: Int J Environ Res Public Health. 2021 May 16;18(10):5289. doi: 10.3390/ijerph18105289 (PMC8157019; doi:10.3390/ijerph18105289)
Supplement: Supplementary file 1 [file ijerph-18-05289-s001.zip › ijerph-1182321-supplementary/Supplementary File/Supplementary Figure 1.pdf]

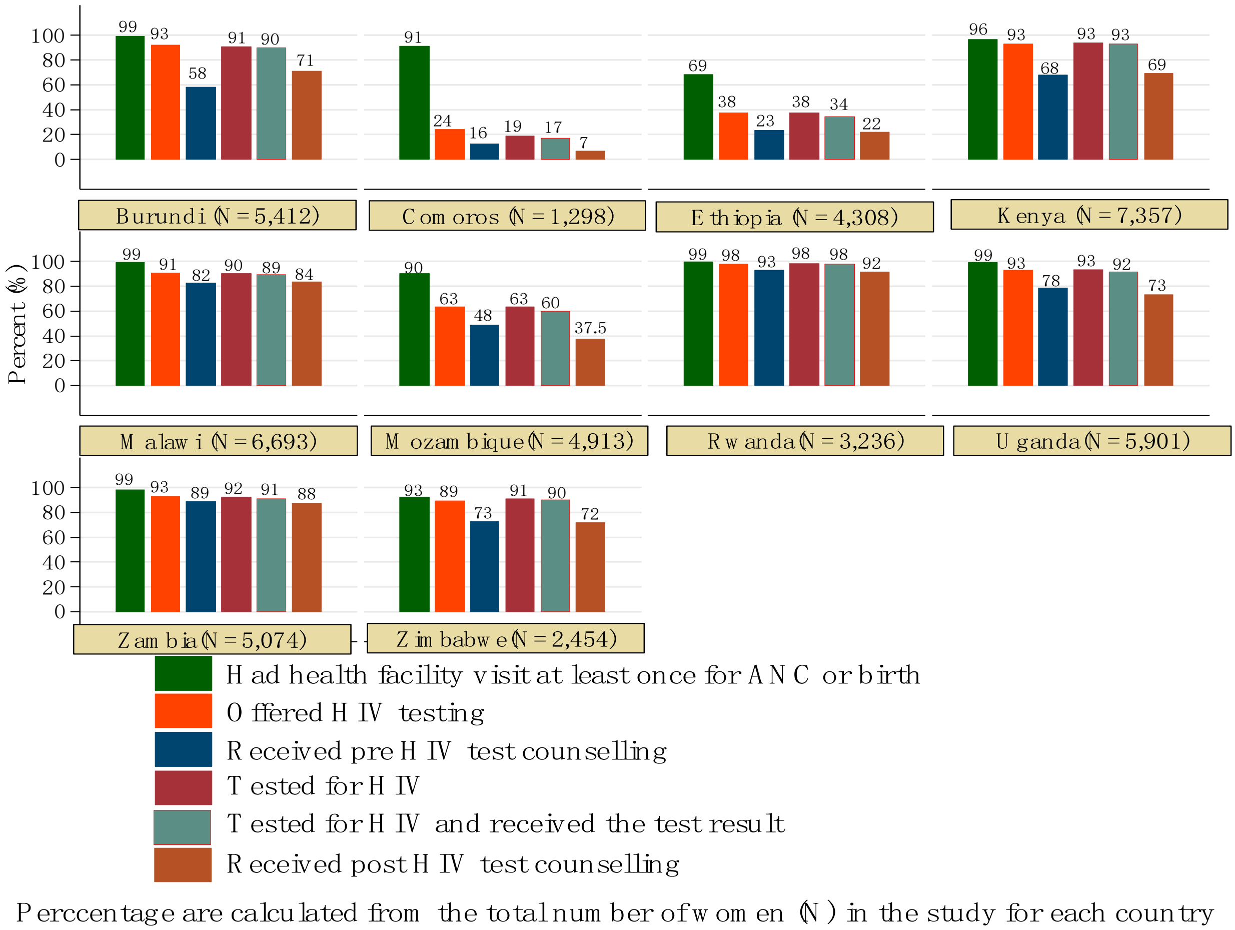

Figure 1: Health facility visit during pregnancy and HIV test services uptake in East Africa countries.
