# Supplementary material for: Prenatal HIV Test Uptake and Its Associated Factors for Prevention of Mother to Child Transmission of HIV in East Africa
Source: Int J Environ Res Public Health. 2021 May 16;18(10):5289. doi: 10.3390/ijerph18105289 (PMC8157019; doi:10.3390/ijerph18105289)
Supplement: Supplementary file 1 [file ijerph-18-05289-s001.zip › ijerph-1182321-supplementary/Supplementary File/Supplementary Table 1.pdf]

Table 1A: Characteristics of the study participants in each and all East African countries (DHS, 2011–2017)

	Burundi	Comoros	Ethiopia	Kenya	Malawi	Mozambique	Rwanda	Uganda	Zambia	Zimbabwe	Total participants
	n (%)	n (%)	n (%)	n (%)	n (%)	n (%)	n (%)	n (%)	n (%)	n (%)	n (%)
**Community-level factors**											
Residence											
Urban	489 (9)	368(28.4)	520(12.1)	2618(35.6)	911(13.6)	1356(27.6)	561(17.3)	1258(21.3)	1711(33.7)	689(28.1)	10481 (22.5)
Rural	4923(91)	929(71.6)	3788(87.9)	4739(64.4)	5781(86.4)	3557(72.4)	2675(82.7)	4643(78.7)	3363(66.3)	1765(71.9)	36164 (77.5)
Predisposing factors											
Demographic Factors											
Maternal age											
15-24	1407(26)	404(31.2)	1260(29.3)	2864(38.9)	3083(46.1)	2015(41.2)	871(26.9)	2511(42.6)	1981(39.1)	966(39.4)	17364 (37.3)
25-34	2742(50.7)	622(47.9)	2189(50.9)	3432(46.7)	2584(38.7)	2000(40.8)	1666(51.5)	2473(41.9)	2194(43.2)	1144(46.6)	21045 (45.1)
35-49	1259(23.3)	271(20.9)	854(19.9)	1061(14.4)	1018(15.2)	882(18)	699(21.6)	912(15.5)	899(17.3)	343(14)	8198 (17.6)
Maternal education											
No education	2365 (43.7)	562(43.3)	2606(60.5)	834(11.3)	794(11.9)	1747(35.6)	439(13.6)	566(9.6)	533(10.5)	32(1.3)	10478 (22.5)
Primary	2398 (45.3)	323(24.9)	1319(30.6)	4023(54.7)	4480(66.9)	2546(51.8)	2316(71.6)	3577(60.6)	2740(54.1)	787(32.1)	24509 (52.6)
Secondary and higher	650 (12)	413(31.8)	383(8.9)	2499(34)	1419(21.2)	620(12.6)	481(14.8)	1757(29.8)	1795(35.4)	1635(66.6)	11652 (24.9)
Maternal occupation											
Not working	412(7.6)	741(59.2)	2510(58.3)	1278(36.2)	2138(32)	2656(81.4)	256(7.9)	1106(18.8)	2251(44.9)	1329(54.3)	14678 (35.7)
Professional work	494(9.1)	193(15.4)	694(16.1)	565(16)	552(8.2)	516(15.8)	426(13.2)	1209(20.5)	1024(20.4)	757(31)	6429 (15.7)
Non-professional work	4506(83.3)	318(25.4)	1104(25.6)	1687(47.8)	4002(59.8)	89(2.8)	2553(78.9)	3583(60.8)	1738(34.7)	360(14.7)	19940 (48.6)
Partner education											
No education	1775(36.2)	459(36.9)	1838(45.1)	304(9.5)	637(11.4)	1376(29.6)	462(16)	458(9.2)	418(9.2)	27(1.3)	7755 (20.4)
Primary	2532(51.5)	322(25.9)	1651(40.5)	1609(50.6)	3018(54)	2412(51.9)	2051(71)	2618(52.7)	1837(40.6)	483(23.1)	18533 (48.6)
Secondary and Higher	605(12.3)	462(37.1)	588(14.4)	1270(39.9)	1935(34.6)	856(18.5)	374(13)	1896(38.1)	2268(50.2)	1582(75.6)	11837 (31.0)
History of sexual violence											
No	2788(75.5)	1012(98.1)	1661(89)	1250(90.3)	1526(81.8)	2519(93.3)	669(92)	2462(78.2)	3225(84.2)	1682(89)	18794 (85.0)
Yes	906(24.5)	20(1.9)	206(11)	134(9.7)	340(18.2)	180(6.7)	59(8)	687(21.8)	604(15.8)	207(11)	3342 (15.0)
Media exposure											
Read newspapers or magazines											
No	5181(95.7)	1105(85.1)	4004(93)	5214(70.9)	5601(83.7)	4412(89.8)	2543(78.7)	4778(81)	3737(73.6)	1606(65.5)	38180 (81.9)
Yes	231(4.3)	193(14.9)	304(7)	2141(29.1)	1091(16.3)	501(10.2)	689(21.3)	1123(19)	1338(26.4)	848(34.5)	8458 (18.1)
Listened to the radio											
No	3045(56.3)	649(50)	3115(72)	1564(21.3)	3569(53.3)	1705(34.7)	600(18.5)	1623(27.5)	2169(42.8)	1094(44.6)	19134 (41.0)
Yes	2367(43.7)	649(50)	1193(28)	5790(78.7)	3124(46.7)	3208(65.3)	2636(81.5)	4278(72.5)	2905(57.2)	1359(55.4)	27508 (59.0)
Watched television											
No	5022(92.8)	444(34.2)	3494(81.1)	4243(57.7)	5656(84.5)	3590(73.1)	2023(62.6)	4288(72.7)	3365(66.3)	1549(63.1)	33673 (72.2)
Yes	390(7.2)	854(65.8)	814(18.9)	3108(43.3)	1037(15.5)	1323(26.9)	1207(37.4)	1614(27.3)	1709(33.7)	905(36.9)	12961 (27.8)
**Enabling factors**											
Household wealth index											
Poor	2387(44.1)	569(43.8)	1011(23.5)	1823(24.8)	3210(48)	2211(45)	1464(45.3)	2578(43.7)	2417(47.6)	1114(45.4)	21187 (45.4)

Middle	2141(39.6)	513(39.6)	2630(61.1)	2792(38)	2427(36.3)	1965(40)	1195(36.9)	1120(19)	1028(20.3)	991(40.4)	17617 (37.8)
Rich	885(16.3)	216(16.6)	667(15.5)	2741(37.2)	1056(15.7)	736(15)	576(17.8)	2203(37.3)	1629(32.1)	349(14.2)	7840 (16.8)
Household Decision making											
Involved	2485(50.7)	296(25.4)	2618(64.3)	1048(35.9)	1953(35.6)	1517(36.8)	1479(56.6)	1563(31.8)	1782(43.8)	1280(61.2)	16020 (44.1)
Not involved	2415(49.3)	870(74.6)	1452(35.7)	1868(64.1)	3528(64.4)	2610(63.2)	1133(43.4)	3352(68.2)	2290(56.2)	810(38.8)	20328 (55.9)
Health facility distance											
Challenging	1793(33.1)	600(46.3)	2606(60.5)	947(26.7)	3830(57.2)	2905(59.1)	745(23)	2407(40.8)	2268(44.7)	968(39.5)	19069 (44.5)
Not challenging	3619(66.9)	698(53.7)	1702(39.5)	2595(73.3)	2863(42.8)	2008(40.9)	2491(77)	3494(59.2)	2807(55.3)	1485(60.5)	23761 (55.5)
Aware perinatal MTCT of HIV											
Aware MTCT during pregnancy											
No	735(14)	484(38.8)	1216(31.2)	3425(46.8)	1278(19.5)	969(20.1)	1014(31.4)	1675(28.4)	1838(36.4)	198(8.2)	12831 (28.1)
Yes	4517(86)	761(61.2)	2676(68.8)	3901(53.2)	5261(80.5)	3834(79.9)	2218(68.6)	4214(71.6)	3216(63.6)	2229(91.8)	32827 (71.9)
Aware MTCT during birth											
No	281(5.4)	530(42.6)	1026(26.4)	1621(22.1)	1018(15.6)	941(19.6)	105(3.3)	398(6.8)	559(11.1)	270(11.1)	6750 (14.8)
Yes	4971(94.6)	715(57.4)	2866(73.6)	5708(77.9)	5520(84.4)	3862(80.4)	3129(96.7)	5491(93.2)	4494(88.9)	2157(88.9)	38914 (85.2)
Aware MTCT during breastfeeding											
No	484(9.2)	469(37.7)	887(22.8)	815(11.1)	608(9.3)	817(17)	166(5.1)	577(9.8)	413(8.2)	363(15)	5600 (12.3)
Yes	4768(90.8)	776(62.3)	3005(77.2)	6514(88.9)	5930(90.7)	3986(83)	3069(94.9)	5312(90.2)	4640(91.8)	2064(85)	40064 (87.7)
**Need factors**											
A desire for the pregnancy											
Wanted pregnancy	4884(90.2)	1176 (90.6)	3943(91.5)	3155(89.1)	5933(88.7)	4750(96.7)	2800(86.6)	5351(90.7)	4751(93.7)	2270(92.5)	39012 (91.1)
Unwanted pregnancy	528(9.8)	122 (9.4)	365(8.5)	387(10.9)	760(11.3)	163(3.3)	433(13.4)	550(9.3)	320(6.3)	184 (7.5)	3812 (8.9)

n= weighted number of women who participated in the study in each and all East African countries
