# Supplementary material for: Prenatal HIV Test Uptake and Its Associated Factors for Prevention of Mother to Child Transmission of HIV in East Africa
Source: Int J Environ Res Public Health. 2021 May 16;18(10):5289. doi: 10.3390/ijerph18105289 (PMC8157019; doi:10.3390/ijerph18105289)
Supplement: Supplementary file 1 [file ijerph-18-05289-s001.zip › ijerph-1182321-supplementary/Supplementary File/Supplementary Table 2.pdf]

Supplementary table 1a: Adjusted OR (95% CI) of factors associated with prenatal HIV test uptake for PMTCT among mother aged 15-49 years in Burundi, Comoros, Ethiopia, Kenya, and Malawi

	Burundi		Comoros		Ethiopia		Kenya		Malawi	
Characteristics	aOR (95% CI)	P-Value	aOR (95% CI)	P-Value	aOR (95% CI)	P-Value	aOR (95% CI)	P-Value	aOR (95% CI)	P-Value
**Community Level Factors**										
Residence										
Urban	1.00		1.00		1.00		1.00		1.00	
Rural	0.61(0.29, 1.28)	0.194	0.83(0.42, 1.66)	0.615	0.38(0.20, 0.73)	0.004	1.05(0.55, 2.01)	0.878	1.12(0.50, 2.51)	0.768
**Predisposing factors**										
**Socio-demographics factors**										
Maternal age										
15-19 years	1.00		1.00		1.00		1.00		1.00	
20-34 years	0.85(0.56, 1.27)	0.435	1.17(0.67, 2.04)	0.567	0.91(0.63, 1.32)	0.642	0.83(0.48, 1.45)	0.528	0.94(0.60, 1.49)	0.812
35-49 years	0.66(0.40, 1.09)	0.112	1.21(0.52, 2.82)	0.653	1.08(0.66, 1.76)	0.741	0.76(0.39, 1.47)	0.425	0.80(0.39, 1.63)	0.539
Maternal education										
No education	1.00		1.00		1.00		1.00		1.00	
Primary	1.17(0.85, 1.62)	0.310	0.72(0.36, 1.46)	0.373	1.89(1.27, 2.81)	0.002	2.06(1.01, 4.21)	0.046	1.08(0.58, 2.02)	0.795
Secondary and higher	1.24(0.67, 2.30)	0.481	1.32(0.69, 2.55)	0.390	2.41(1.18, 4.94)	0.016	7.81(1.86, 32.8)	0.005	0.98(0.42, 2.28)	0.972
Maternal occupation										
Not working	1.00		1.00		1.00		1.00		1.00	
Professional work	0.66(0.29, 1.53)	0.341	0.60(0.32, 1.12)	0.112	1.13(0.67, 1.91)	0.638	0.71(0.29, 1.77)	0.474	1.35(0.54, 3.35)	0.510
Nonprofessional work	0.81(0.40, 1.62)	0.557	0.73(0.38, 1.42)	0.363	1.27(0.87, 1.85)	0.213	1.36(0.78, 2.35)	0.271	0.94(0.61, 1.47)	0.817
Partner education										
No education	1.00		1.00		1.00		1.00		1.00	
Primary	0.86(0.63, 1.19)	0.382	2.19(1.20, 3.98)	0.010	1.13(0.78, 1.63)	0.499	2.46(1.17, 5.17)	0.017	1.21(0.63, 2.31)	0.550
Secondary and Higher	0.86(0.44, 1.69)	0.677	2.72(1.37, 5.37)	0.004	1.33(0.74, 2.37)	0.330	1.78(0.75, 4.20)	0.184	1.17(0.54, 2.52)	0.672
History of Sexual violence										
No	1.00		1.00		1.00		1.00		1.00	
Yes	0.65(0.48, 0.89)	0.007	0.39(0.03, 4.74)	0.464	1.31(0.71, 2.41)	0.373	1.03(0.43, 2.48)	0.930	0.79(0.48, 1.29)	0.348
Media exposure										
Read newspapers or magazines										
No	1.00		1.00		1.00		1.00		1.00	
Yes	0.99(0.42, 2.30)	0.987	1.78(0.94, 3.37)	0.075	1.34(0.60, 2.98)	0.462	1.73(0.70, 4.26)	0.229	1.02(0.57, 1.82)	0.923
Listened to the radio										
No	1.00		1.00		1.00		1.00		1.00	
Yes	1.31(0.94, 1.83)	0.110	0.83(0.48, 1.41)	0.493	1.08(0.74, 1.58)	0.656	1.34(0.79, 2.27)	0.272	1.43(0.95, 2.17)	0.082

Watched television										
No	1.00		1.00		1.00		1.00		1.00	
Yes	0.83(0.40, 1.73)	0.627	1.10(0.59, 2.04)	0.751	1.83(1.14, 2.92)	0.011	2.32(0.88, 6.10)	0.088	0.87(0.47, 1.63)	0.676
**Enabling factors**										
Household wealth index										
Poor	1.00		1.00		1.00		1.00		1.00	
Middle	1.05(0.76, 1.45)	0.773	1.25(0.68, 2.30)	0.452	2.36(1.48, 3.75)	<0.001	1.22(0.59, 2.53)	0.579	0.86(0.56, 1.31)	0.486
Rich	1.57(0.81, 3.04)	0.180	0.77(0.33, 1.81)	0.563	2.03(1.87, 3.74	<0.001	1.85(0.34, 9.81)	0.469	1.26(0.52, 3.02)	0.595
Household Decision making										
Not Involved	1.00		1.00		1.00		1.00		1.00	
Involved	0.95(0.71, 1.28)	0.776	1.28(0.74, 2.20)	0.365	0.76(0.54, 1.07)	0.121	2.03(1.22, 3.38)	0.006	0.72(0.47, 1.09)	0.128
Health facility distance										
A big problem	0.84(0.60, 1.16)	0.283	0.77(0.46, 1.31)	0.330	0.87(0.61, 1.23)	0.425	0.72(0.42, 1.25)	0.235	1.17(0.74, 1.85)	0.511
Not a big problem	1.00		1.00		1.00		1.00		1.00	
Perinatal awareness of PMTCT of HIV										
Aware of MTCT during pregnancy										
No	1.00		1.00		1.00		1.00		1.00	
Yes	1.07(0.72, 1.59)	0.725	1.21(0.63, 2.31)	0.558	1.22(0.78, 1.90)	0.364	0.46(0.26, 0.81)	0.007	1.27(0.67, 2.39)	0.459
Aware of MTCT during birth										
No	1.00		1.00		1.00		1.00		1.00	
Yes	1.10(0.60, 2.00)	0.744	1.11(0.58, 2.15)	0.733	1.34(0.81, 2.24)	0.249	2.86(1.60, 5.11)	<0.001	1.56(0.73, 3.35)	0.246
Aware of MTCT during breastfeeding										
No	1.00		1.00		1.00		1.00		1.00	
Yes	1.33(0.81, 2.17)	0.249	0.74(0.35, 1.55)	0.425	1.47(0.89, 2.43)	0.131	2.29(1.24, 4.20)	0.007	0.92(0.40, 2.10)	0.845
Need factors										
Desire for pregnancy										
Desired pregnancy	1.00		1.00		1.00		1.00		1.00	
Not desired pregnancy	0.97(0.57, 1.62)	0.912	1.17(0.59, 2.34)	0.636	0.67(0.33, 1.36)	0.274	0.54(0.25, 1.17)	0.120	0.51(0.29, 0.87)	0.014

Supplementary table 1b: Adjusted OR (95% CI) of factors associated with prenatal HIV test uptake for PMTCT among mothers aged 15-49 years in Mozambique, Rwanda, Uganda, Zambia, and Zimbabwe.

	**Mozambique**		**Rwanda**		**Uganda**		**Zambia**		**Zimbabwe**	
Characteristics	aOR (95% CI)	P-Value	aOR (95% CI)	P-Value	aOR (95% CI)	P-Value	aOR (95% CI)	P-Value	aOR (95% CI)	P-Value
**Community-Level Factors**										
Residence										
Urban	1.00		1.00		1.00		1.00		1.00	
Rural	1.28(0.72, 2.27)	0.383	0.47(0.06, 3.60)	0.469	0.56(0.26, 1.19)	0.133	0.55(0.32, 0.93)	0.027	2.07(0.66, 6.51)	0.209
**Predisposing factors**										
**Socio-demographics factors**										
Maternal age										
15-19 years	1.00		1.00		1.00		1.00		1.00	
20-34 years	1.12(0.78, 1.60)	0.528	1.93(0.41, 9.01)	0.401	1.24(0.87, 1.78)	0.218	1.00(0.70, 1.42)	0.993	0.61(0.37, 1.01)	0.060
35-49 years	1.48(0.90, 2.45)	0.119	2.87(0.38, 21.60)	0.304	0.65(0.41, 1.04)	0.079	0.85(0.53, 1.37)	0.517	0.45(0.23, 0.90)	0.024
Maternal education										
No education	1.00		1.00		1.00		1.00			
Primary	1.21(0.85, 1.72)	0.286	3.05(0.71, 13.11)	0.133	1.20(0.75, 1.92)	0.435	1.65(1.11, 2.45)	0.013	****	
Secondary and higher	1.89(0.84, 4.27)	0.122	2.64(0.38, 18.24)	0.323	3.16(1.50, 6.65)	0.003	1.58(0.89, 2.82)	0.114		
Maternal occupation										
Not working	1.00				1.00		1.00		1.00	
Professional work	0.80(0.47, 1.35)	0.409	****		1.26(0.70, 2.25)	0.423	0.83(0.52, 1.33)	0.448	0.48(0.27, 0.86)	0.014
Nonprofessional work	3.26(0.60, 17.66)	0.170			1.23(0.81, 1.88)	0.318	0.74(0.53, 1.05)	0.100	0.58(0.26, 1.25)	0.166
Partner education										
No education	1.00		1.00		1.00		1.00		1.00	
Primary	1.55(1.09, 2.20)	0.014	3.32(0.76, 14.40)	0.109	0.96(0.55, 1.67)	0.899	0.88(0.55, 1.42)	0.619	14.3(1.94, 105.5)	0.009
Secondary and Higher	1.66(0.92, 3.02)	0.091	3.27(0.30, 35.26)	0.327	1.61(0.87, 2.94)	0.122	1.14(0.65, 2.00)	0.629	16.0(2.20, 116.7)	0.006
History of Sexual violence										
No	1.00		1.00		1.00		1.00		1.00	
Yes	1.48(0.80, 2.71)	0.203	0.88(0.08, 9.51)	0.916	0.94(0.65, 1.38)	0.786	1.13(0.76, 1.67)	0.530	0.57(0.24, 1.35)	0.207
Media exposure										
Read newspapers or magazines										
No	1.00		1.00		1.00		1.00		1.00	
Yes	1.13(0.55, 2.32)	0.719	3.43(0.39, 29.77)	0.262	1.30(0.70, 2.41)	0.389	1.46(0.92, 2.30)	0.103	1.08(0.59, 1.99)	0.786
Listened to the radio										
No	1.00		1.00		1.00		1.00		1.00	
Yes	1.00(0.70, 1.44)	0.962	0.70(0.15, 3.31)	0.659	1.24(0.87, 1.76)	0.231	0.98(0.73, 1.32)	0.925	1.14(0.61, 2.16)	0.666
Watched television										
No	1.00		1.00		1.00		1.00		1.00	
Yes	1.67(0.92, 3.04)	0.088	0.56(0.14, 2.18)		0.97(0.60, 1.56)	0.912	1.17(0.76, 1.79)	0.454	1.70(0.75, 3.85)	0.199
**Enabling factors**										

Household wealth index										
Poor	1.00		1.00		1.00		1.00		1.00	
Middle	1.63(1.03, 2.58)	0.036	1.71(0.31, 9.27)	0.527	1.55(1.03, 2.35)	0.034	1.05(0.72, 1.53)	0.768	1.47(0.80, 2.68)	0.204
Rich	3.15(1.39, 7.10)	0.006	3.71(0.33, 41.8)	0.287	0.96(0.41, 2.25)	0.938	0.95(0.37, 2.44)	0.923	2.48(0.73, 8.38)	0.140
Household Decision making										
Not Involved	1.00		1.00		1.00		1.00		1.00	
Involved	1.16(0.82, 1.63)	0.388	0.78(0.19, 3.18)	0.732	0.96(0.69, 1.34)	0.838	0.82(0.60, 1.12)	0.220	0.56(0.31, 1.01)	0.057
Health facility distance										
Challenging	1.26(0.79, 2.00)	0.331	0.19(0.04, 0.96)	0.044	1.15(0.82, 1.61)	0.421	0.90(0.63, 1.26)	0.531	1.08(0.56, 2.08)	0.811
Not challenging	1.00		1.00		1.00		1.00		1.00	
Perinatal awareness of PMTCT of HIV										
Aware of MTCT during pregnancy										
No	1.00		1.00		1.00		1.00		1.00	
Yes	0.76(0.48, 1.20)	0.253	8.33(2.39, 29.01)	0.001	0.56(0.37, 0.83)	0.004	0.90(0.65, 1.22)	0.508	0.57(0.25, 1.31)	0.191
Aware of MTCT during birth										
No	1.00		1.00		1.00		1.00		1.00	
Yes	1.45(0.93, 2.26)	0.092	2.58(0.15, 42.8)	0.506	1.50(0.83, 2.71)	0.174	2.43(1.64, 3.60)	<0.001	5.89(2.72, 12.74)	<0.001
Aware of MTCT during breastfeeding										
No	1.00		1.00		1.00		1.00		1.00	
Yes	3.13(1.89, 5.20)	<0.001	0.47(0.006, 33.3)	0.732	1.19(0.69, 2.06)	0.515	1.71(1.09, 2.67)	0.018	1.13(0.59, 2.16)	0.691
**Need factors**										
A desire for the pregnancy										
Wanted pregnancy	1.00		1.00		1.00		1.00		1.00	
Unwanted pregnancy	0.58(0.25, 1.33)	0.202	0.07(0.01, 0.45)	0.005	0.94(0.53, 1.67)	0.859	0.76(0.41, 1.42)	0.405	0.069(0.008, 0.46)	0.006

***** no estimate due to small sample size or empty cell
